# Psychological Distress in Out-Patients Assessed for Chronic Pain Compared to Those with Rheumatoid Arthritis

**DOI:** 10.1155/2016/7071907

**Published:** 2016-03-01

**Authors:** D. Rice, S. Mehta, A. Shapiro, J. Pope, M. Harth, P. Morley-Forster, K. Sequeira, R. Teasell

**Affiliations:** ^1^Aging, Rehabilitation, and Geriatric Care, Lawson Health Research Institute, London, ON, Canada N6C 0A7; ^2^Parkwood Institute, London, ON, Canada N6C 0A7; ^3^Western University, London, ON, Canada N6A 3K7; ^4^St. Joseph's Health Care London, London, ON, Canada N6C 0A7

## Abstract

*Background*. Patients diagnosed with chronic pain (CP) and rheumatoid arthritis (RA) represent two samples with overlapping symptoms, such as experiencing significant pain.* Objectives*. To compare the level of psychological distress among patients diagnosed CP attending a specialist pain clinic with those attending a specialist RA clinic.* Measures*. A cross-sectional study was conducted at an academic specialist chronic pain and rheumatology clinic.* Participants*. 330 participants included a CP group (*n* = 167) and a RA group (*n* = 163) completed a booklet of questionnaires regarding demographic characteristics, duration, and severity of their pain. Psychological and personality variables were compared between the CP and RA participants using a Multivariate Analysis of Covariance (MANCOVA).* Results*. Level of psychological distress based on the subscales of the DASS (depression, anxiety, and stress), PASS (escape avoidance, cognitive anxiety, fear of pain, and physiological anxiety), and PCS (rumination, magnification, and helplessness) was significantly higher in the CP group compared to the RA group. Categorization of individuals based on DASS severity resulted in significant differences in rates of depression and anxiety symptoms between groups, with a greater number of CP participants displaying more severe depressive and anxiety symptoms.* Discussion and Conclusions*. This study found greater levels of psychological distress among CP individuals referred to an academic pain clinic when compared to RA patients referred to an academic rheumatology clinic.

## 1. Introduction

Chronic pain (CP) is a common disorder affecting up to 35% of individuals [1] and is a common cause of job related disability and missed work [2]. A multicentre Canadian study (STOP-PAIN), based on 8 tertiary pain clinics, found that over 66% patients in the study experienced high levels of pain burden, sleep disturbance, and interference with normal activities of daily living [3]. The group also determined median direct (e.g., drug treatment) and indirect costs (e.g., lost labour time) per participant was $1,462 per month [4].

One potential reason for the high personal and economic costs related to CP is the high prevalence of psychological distress among these individuals. Psychological factors are widely believed to play a significant role in CP [5]. Preexisting depression, anxiety, and stress may predispose some individuals to progress to a CP condition, while CP in turn leads to anxiety and/or depression, resulting in a so-called vicious cycle [6]. Over 50% of patients in tertiary Canadian pain clinics experience moderate to severe levels of depression and anxiety [3].

Most individuals with rheumatoid arthritis (RA) also experience CP. Andersson et al. [7] found that only 20% of patients reported not having CP within 5 years of RA diagnosis. Much like CP, the pain associated with RA leads to activity limitations and has a significant impact on patients' quality of life [8]. The QUEST-RA study examined data from 32 countries and found that 37% of individuals reported work disability due to RA [9].

However, what distinguishes RA from chronic soft tissue pain disorders is the presence versus the absence of radiographically demonstrable structural abnormalities accounting for the pain. Pain among individuals diagnosed with RA is easier to define physically and pathophysiologically, through affected joint activity (swelling, inflammation) and damage (i.e., subluxations, loss of cartilage, and bony erosions). Difficulties arise with regional or general musculoskeletal pain syndromes of uncertain etiology because they do not readily lend themselves to such structural diagnoses and hence specific treatment through the acute medical model. This becomes problematic when the patient is, in many cases, held responsible for his or her own pain. This is revealed when patients receive diagnoses such as malingering, hysteria, somatoform pain disorder, secondary gain, or litigation neurosis whereby the patient is seen as exaggerating their symptoms for their own personal gain rather than the pain symptoms being associated to legitimate pain [10]. A patient with nonspecific low back pain that does not have specific radiographic abnormalities is never to be considered truly disabled even though the clinical presentation may be virtually identical to that of someone “legitimately” disabled with recognizable radiological abnormalities [11]. A qualitative study found that patients with CP commonly reported feeling stressed from interacting with health professionals as they believed that their doctors thought the patients' pain was not real and instead was a result of psychological problems [12]. Alternatively, those with RA feel supported and “believed” by their doctors as they have a diagnosis which verifies the cause of their pain [12].

Furthermore, though both groups of patients experience increased levels of disability compared to the general population, access times to receive specialised services differ. Peng et al. [13] found that the median wait time to gain initial consult at a multidisciplinary pain treatment facility was 6 months, with wait times of up to 5 years for specialised treatments. In contrast, Jamal et al. [14] surveyed practicing rheumatologists in the Greater Toronto Area (GTA) and found that most individuals with RA were seen by a specialist within 3–6 months of their symptoms. A systematic review found that wait times greater than 6 months for CP management resulted in deterioration of health-related quality of life and psychological well-being among these individuals [15].

Previous studies have compared patients with documented sources of pain to those whose etiology of pain is less certain through samples comprised of patients with CP and RA. Generally, these studies have found significant differences between samples, whereby patients with CP experience greater pain and psychological distress than those with a diagnosis of RA; however, the increased psychological distress may be a result of the greater pain that patients diagnosed with CP report [8, 16].

The potential lack of clear structural pathophysiological abnormalities among individuals experiencing chronic soft tissue pain along with longer wait times to be seen by a specialist may result in patients with CP experiencing greater psychological distress than those with RA. Current literature in this field often looks at one sample of patients (either those diagnosed with CP or RA) and considers a limited number of psychological variables. The current study aimed to compare patients with CP directly to those diagnosed with RA while testing a broader range of psychological variables while accounting for differences in pain. The current study hypothesizes that patients with CP will experience greater psychological distress compared to patients diagnosed with RA, even after controlling for pain intensity and demographic variables. Hence, the objective of the current study was to compare the level of psychological distress among individuals with CP attending a specialist pain clinic to patients attending a specialist RA clinic.

## 2. Methods

### 2.1. Participants

Participants included two samples of patients, a CP group and a RA group. Patients were recruited over a period of 20 months from academic hospitals in London, Ontario. Patients with CP were recruited from an academic specialist chronic pain clinic, while patients with RA were recruited from an academic specialist rheumatology clinic. Participants with CP were included if they experienced pain for at least 3 months. Diagnosis of CP and RA was conducted by a specialist physician. Eligible participants from both study groups signed informed consent. Ethics was reviewed and approved by the Office of Research Ethics at Western University in London, Ontario, Canada.

### 2.2. Procedures

Researchers followed a similar protocol for eligible CP and RA participants. Two weeks prior to in-person clinic appointments, packages containing the study information letter, a consent form, and the first of two questionnaire booklets were mailed to each prospective CP and RA participant. Research assistants contacted patients via telephone to confirm their interest in participating and to provide answers to patients' questions. Consenting participants completed a booklet of questionnaires regarding demographic characteristics and cause, duration, and severity of their pain.

### 2.3. Outcome Measures

#### 2.3.1. Depression Anxiety Stress Scales-Short Form (DASS-SF)

The DASS-SF is a 21-item self-report questionnaire yielding separate scores for depression, anxiety, and stress over the previous week. The items are scored on a 4-point scale (0 = “did not apply to me at all” to 3 = “applied to me very much or most of the time”) where higher scores indicate greater levels of distress [17]. The DASS-SF measure is reliable and valid [18] and correlates highly with the original 42-item DASS [17]. The following cut-off scores have been recommended for each subscale: depression (0–4 = normal, 5-6 = mild, 7–10 = moderate, 11–13 = severe, and ≥14 = extremely severe), anxiety (0–3 = normal, 4-5 = mild, 6-7 = moderate, 8-9 = severe, and ≥10 = extremely severe), and stress (0–7 = normal, 8-9 = mild, 10–12 = moderate, 13–16 = severe, and ≥17 = extremely severe). Cronbach's alpha within the CP sample was 0.86 for the stress subscale, 0.80 for the anxiety subscale, and 0.89 for the depression subscale. Cronbach's alpha within the RA sample was 0.82 for the stress subscale, 0.81 for the anxiety subscale, and 0.78 for the depression subscale.

#### 2.3.2. Acceptance and Action Questionnaire (AAQ)

The AAQ is a 9-item self-report measure of experiential avoidance, that is, unwillingness to remain in contact with distressing private experiences (body sensations, emotions, and thoughts) and the inclination to alter the form or frequency of these experiences [19]. Respondents rate the degree to which items apply to them on a 7-point scale ranging from 1 (“never true”) to 7 (“always true”). It yields a single factor solution and is correlated with a wide range of negative behavioural and physical health outcomes [19]. Cronbach's alpha was 0.67 for patients with CP and 0.69 for patients with RA in the present study.

#### 2.3.3. Pain Anxiety Symptom Scale (PASS-20)

The PASS-20 is designed to measure fear of pain. The PASS-20 consists of 4 subscales include avoidance, cognitive anxiety, fearful thinking, and physiological anxiety. These subscales include 5 items each where each item is rated on a frequency scale from “never” (0) to “always” (5) with scores ranging from 0 to 100. The PASS-20 has demonstrated good psychometric properties [20]. Cronbach's alpha for patients with CP was 0.76 for the avoidance subscale, 0.85 for the cognitive anxiety subscale, 0.82 for the fearful thinking subscale, and 0.66 for the physiological anxiety subscale. Cronbach's alpha for patients with RA was 0.75 for the avoidance subscale, 0.84 for the cognitive anxiety subscale, 0.64 for the fearful thinking subscale, and 0.80 for the physiological anxiety subscale.

#### 2.3.4. Pain Catastrophizing Scale (PCS)

The PCS contains 13 items assessing the tendency to misinterpret and exaggerate the threat value of pain sensations. It has good psychometric properties and includes 3 main factors: rumination, magnification, and helplessness [21]. The PCS asks participants to reflect on past painful experiences and to indicate the degree of experienced thoughts and feelings on a 5-point scale (0 = not at all, 4 = all the time). Cronbach's alpha for patients with CP was 0.86 for the rumination subscale, 0.74 for the magnification subscale, and 0.89 for the helplessness subscale. Cronbach's alpha for patients with RA was 0.66 for the rumination subscale, 0.73 for the magnification subscale, and 0.90 for the helplessness subscale.

#### 2.3.5. Average Pain Intensity Rating

Pain ratings for current, least, average, and worst pain were summed to yield an aggregate pain intensity score. The scale ranges from 0 to 10 with 0 indicating no pain and 10 indicating intense pain. This composite pain intensity score has been shown to be a very reliable measure of pain intensity in chronic pain patients and has been used in recent research [22]. Cronbach's alpha was 0.86 for patients with CP and 0.93 for patients with RA in the present study.

### 2.4. Statistical Analysis

Descriptive analysis was performed to compare baseline demographic factors between the groups. Psychological and personality variables (DASS-SF, AAQ, PCS, and PASS) were compared between the CP and RA participants using a Multivariate Analysis of Covariance (MANCOVA) with Bonferroni adjustment for multiple comparisons and associated effect sizes were calculated. Age, gender, and pain intensity were included in the model as covariates. Patients with missing data necessary for analysis were excluded by case listwise. Preliminary tests were conducted to confirm there was no violation of MANCOVA assumptions. The homogeneity of variance assumption was violated for a number of variables so a more conservative alpha level of 0.01 was set in order to determine significance in the univariate *F*-Test [23]. No other assumptions tested were violated within our sample. Individuals were then categorized based on DASS severity levels of depression, anxiety, and stress symptoms according to the recommended ranges provided by DASS and frequencies were compared between patients with CP and RA.

## 3. Results

**Figure 1:**
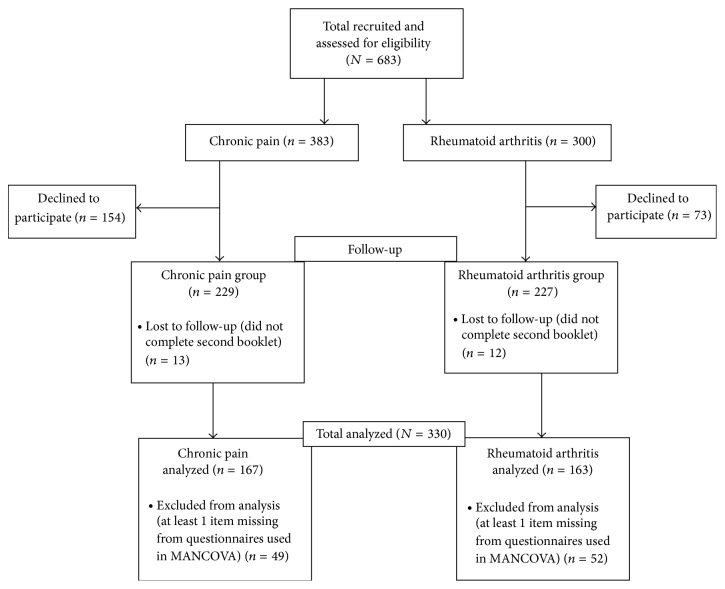
Participant flow chart.

Overall, 683 eligible patients were approached; 456 participants consented for this study. A total of 126 participants were excluded for the MANCOVA because they had incomplete measures that were necessary for analysis (see Figure 1). This resulted in a final sample size of 330 study participants from the RA (*n* = 163) and CP (*n* = 167) groups used for analysis. The impact of missing values was not significant. Chi squared results indicated that values were missing completely at random (*χ* ^2^ = 369.34, df = 344, and *p* = 0.166). The 330 study participants included 230 (70%) females and the mean age was 49.9 years (SD = 13.3). Level of education was similar in both groups. However, CP participants had almost double the average pain intensity as the RA participants with a significant difference between groups (*t*(328) = 11.21, *p* < 0.001). Demographic variables for each group are provided in Table 1.

After adjusting for age, gender, and pain intensity, significant differences (*p* < 0.001) were found between groups for AAQ, DASS, PASS, and PCS, with CP participants scoring significantly higher than participants with RA on each measure (Table 2). Scores on the subscales of the DASS (depression, anxiety, and stress), PASS (escape avoidance, cognitive anxiety, fear of pain, and physiological anxiety), and PCS (rumination, magnification, and helplessness) were also significantly higher in the CP group compared to the RA group (*p* < 0.001). Effect sizes based on Cohen's *d* were calculated and ranged from 0.33 to 0.86 representing small (0.20–0.49), medium (0.50–0.79), and large effects (≥0.80) found. The greatest effect sizes were found for the PASS cognitive anxiety subscale (0.86) and the PCS helplessness subscale (0.71) while on average most effect sizes were medium (see Table 2).

Categorization of individuals based on DASS severity demonstrated that a greater number of participants with CP displayed more severe depressive and anxiety symptoms. Increased rates of individuals with mild, moderate, severe, and extremely severe depression symptoms among CP patients were found (16.2%, 28.1%, 13.8%, and 14.4%) compared to RA patients (12.3%, 7.4%, 2.5, and 1.8%). Mild to moderate levels of anxiety were greater among RA patients compared to CP patients (19.6% versus 13.2%, 16.0% versus 11.4%, resp.), while the percentage of those individuals with severe and extremely severe anxiety symptoms was higher in the CP group compared to those with RA (19.2% versus 4.9%, 38.3% versus 9.8%). CP patients had more mild, moderate, severe, and extremely severe stress (12.6%, 18.6%, 13.8%, and 6.0%) than RA patients (3.7%, 4.3%, 1.8%, and 0.6%). Based on our two study populations, individuals with CP experienced more severe depressive, anxiety, and stress symptoms compared to those with chronic RA.

## 4. Discussion

This study compares the experience of pain and psychological distress among individuals with RA compared to CP. Average pain intensity was significantly greater among CP participants than RA, with CP participants experiencing a mean pain intensity almost twice as high as RA patients. CP patients also experienced significantly more psychological distress even after controlling for age, sex, and average pain intensity. The differences between patient samples resulted in medium effect sizes, on average.

Both patients diagnosed with CP and RA have been shown to experience more psychological distress than the general population [24]. Specifically, our study found that CP patients scored significantly higher on AAQ, DASS-SF, PASS-20, and PCS when compared to RA patients. The significantly higher AAQ scores among CP patients compared to RA in the current study indicate more experiential avoidance and psychological inflexibility, which has been negatively associated with pain acceptance [24]. Further, McCracken and Zhao-O'Brien [25] found that there was more acceptance of pain among individuals with CP seen in a primary care setting when compared to a specialty treatment centre. Hence, the increased experiential avoidance scores among CP patients in the current study may be due to our sample representing a more psychologically distressed population from a specialty care clinic.

Significantly higher scores reflecting large and medium effect sizes in PASS cognitive anxiety and PCS among CP patients compared to those with RA suggest the use of different cognitive processing of pain among these individuals. Similarly, Gil et al. [16] reported that patients who endorsed more frequent negative self-statements and negative social cognitions were more likely to have severe pain and psychological distress. It has been suggested that patients with CP may have a cognitive processing bias where they selectively process pain and illness related stimuli [26]. In a study examining the difference in negative thoughts among CP, RA, and individuals with sickle cell anemia, patients with CP had more negative self-statements and social cognitions than patients with RA and sickle cell anemia. The use of therapies that involve cognitive restructuring and mindfulness such as mindfulness-based cognitive therapy may be particularly useful among the CP population by helping to improve psychological distress and the cognitive aspect of the pain experience.

Categorization of the groups based on DASS severity revealed a higher frequency of patients with CP reporting a greater presence of depressive, anxiety, and stress symptoms. There was an absence of depressive symptoms in greater than 75% of RA patients, while only 28% of CP patients had no symptoms. Similarly, almost half the RA patients did not present with anxiety symptoms whereas only 18% of CP patients did not report anxiety symptoms. Symptoms of mild to extremely severe stress were present in only 10% of RA patients while almost 50% of CP patients reported at least mild stress. This marked difference regarding the presence of psychological distress between the two populations may in part be related to the inadequacy of treatment services available for the CP population relative to RA, although to what degree, if any, is unknown.

It is unclear as to the causative relationship between pain and psychological distress in the two populations examined. CP patients may be less likely to be referred to a tertiary care clinic until pain becomes more intense and psychological distress more problematic, while RA patients tend to be referred to specialist rheumatology clinics at any symptom level [14]. Hence, some of the differences seen may be accounted for by a referral bias. CP patients may experience more pain intensity and psychological distress after they have experienced unsuccessful treatment plans from health care professionals [16]. Psychological distress is associated with poor physical outcomes such as functional disability and pain [27] although the relationship is undoubtedly complex. Tang et al. [28] found the presence of mental defeat was a significant predictor of functional disability and distress among individuals with CP. This may result in a cyclical experience of distress and pain, especially for CP patients who demonstrated increased distress when compared to RA patients. The subsequent psychological symptoms may impact the patients' functional disability, further negatively influencing their pain and psychological state.

The uncertainty of a diagnosis for CP may also contribute to the increased psychological distress among CP participants compared to RA participants where a discernible organic pathology is present and can be better quantified. Chibnall et al. [29] found that between-physician consistency was very low for the diagnosis of CP. Due to the lack of specific diagnostic tests to assess the organic pathology of CP, patients may even be labelled as somatizers or even malingerers [30, 31]. Chronic pain patients often feel that the absence of a discernible diagnosis implies that health professionals feel that the pain may just be in their minds [32]. This stigmatization may result in increased distress for the individual, which may further impact their psychological state and ability to function in daily activities. Additionally, Liang et al. [33] found the majority of RA patients were satisfied with the relationship they had with their physicians with only 21% feeling that their doctor “could do more to understand their illness.” This is in marked contrast to CP patients who frequently complain that physicians fail to acknowledge or understand their symptoms.

The current study has a number of limitations. The CP population studied came from a tertiary care clinic and may not be representative of CP patients in general although they would be representative of CP patients referred to specialists. Similarly, the RA population consisted of those seen in a specialist clinic with RA that could have been active or in remission when entered into this study. The current study did not investigate all variables that could account for the difference between the two samples. Factors such as duration of pain, disability, current employment, current litigation or workers compensation status, and interference with sleep may have a strong influence on pain intensity and psychological distress. The current study design did not allow for the examination of a causal relationship among the various variables examined. Patients willing to participate were studied cross-sectionally just before and then at their clinic appointment so cause and effect cannot be determined.

Despite these limitations, results from the current study have several clinical implications. The high levels of depressive and anxiety symptoms among individuals in both populations demonstrate the need for multidisciplinary pain management, in particular psychological counselling and support, which have previously shown to be effective [34]. The difference in symptoms of psychological distress between the two populations may speak towards a lack of sufficient services available for the CP population when compared to patients with RA. Hence, an analysis of the current resources available for the two populations with regard to information and counselling support may help to better understand the differential accessibility to services between the two populations, which potentially lead to differences in psychological distress.

## 5. Conclusion

The current study looked at a variety of psychological variables among individuals with CP referred to an academic pain clinic compared to patients with RA referred to an academic rheumatology clinic. The measures studied included self-reported experiential avoidance, mood, fear of pain, and pain catastrophizing and among each of these variables, significant differences were found with patients diagnosed with CP scoring significantly higher on all psychological measures even after controlling for age, gender, and average pain intensity. The differences between samples based on psychological distress resulted in a number of medium effect sizes with the largest differences seen for measures of pain anxiety and pain catastrophizing. Individuals with CP were also more likely to exhibit severe to extremely severe symptoms of depression and anxiety compared to RA patients. An increased level of distress may serve to heighten chronic pain leading to a cyclical experience of distress and pain. This study points to the importance of psychological distress in both CP and RA patients in their experience of pain. Increased access to multidisciplinary services may be important in managing CP population.

## Figures and Tables

**Table 1:** Sample demographic profile.

Participant characteristics	Tertiary chronic pain	Rheumatoid arthritis
*N*	167	163
Gender		
Male	35.8%	23.8%
Female	64.2%	76.2%
Mean age (SD)	44.49 (10.84)	55.48 (13.38)
Mean years of education (SD)	13.45 (2.72)	13.18 (3.28)
Current relationship status		
Single	12.8%	11.1%
Married	59.8%	67.6%
Divorced	8.7%	4.0%
Separated	3.2%	1.3%
Widowed	2.3%	8.9%
Serious relationship	13.2%	7.1%
Current employment status		
Full-time	13.1%	30.2%
Part-time	5.2%	11.6%
Not working	80.3%	42.2%
Retired	1.3%	16.0%
Average pain intensity (SD)	6.17 (1.88)	3.64 (2.23)

**Table 2:** Pairwise comparison psychological factors by group after controlling for average pain intensity.

Factor	CP mean (SD)	RA mean (SD)	SE	*F*	*p*	*d*	Effect size
AAQ	32.48 (8.17)	27.93 (7.74)	1.02	10.44	<0.001	0.35	Small
DASS depression	7.87 (5.09)	3.15 (3.13)	0.52	37.20	<0.001	0.66	Medium
DASS anxiety	8.37 (4.67)	4.17 (3.61)	0.51	26.54	<0.001	0.56	Medium
DASS stress	8.12 (3.57)	4.85 (3.35)	0.52	35.36	<0.001	0.65	Medium
PASS escape avoidance	14.01 (5.31)	9.44 (5.67)	0.68	16.08	<0.001	0.44	Small
PASS cognitive anxiety	15.75 (5.14)	8.56 (5.73)	0.67	61.68	<0.001	0.86	Large
PASS fear of pain	9.50 (5.91)	4.75 (6.04)	0.75	17.97	<0.001	0.46	Small
PASS physiological anxiety	10.91 (7.70)	3.68 (4.34)	0.78	40.72	<0.001	0.70	Medium
PCS rumination	13.11 (4.03)	9.51 (5.48)	0.59	9.54	<0.001	0.34	Small
PCS magnification	7.34 (3.02)	5.00 (2.18)	0.33	27.02	<0.001	0.57	Medium
PCS helplessness	14.70 (5.16)	9.18 (3.88)	0.56	42.38	<0.001	0.71	Medium
